# Supplementary material for: Deletion of wfs1 Impairs Oligodendrocyte Precursor Cells Dorsal Distribution and Myelination Through the wfs1-hmgcs1 Axis in Zebrafish
Source: Biology (Basel). 2026 Aug 16;15(16):1402. doi: 10.3390/biology15161402 (PMC13510151; doi:10.3390/biology15161402)
Supplement: Supplementary file 1 [file biology-15-01402-s001.zip › File S1-Transcriptome sequencing data and differentially expressed genes (DEGs) in wfs1-- zebrafish at 5 dpf/2.KEGG/wfs1KOvsWT/UP/src/dre03013.html]

dre03013


- dre:324342

- Up regulated genes

324342\_1(3.3994460172095)
- dre:407710

- Up regulated genes

407710(2.19508023604394)
- dre:333980

- Up regulated genes

333980(1.04245960051675)

- dre:393973

- Up regulated genes

393973(1.85263649603668)

- dre:321744

- Up regulated genes

321744\_1(1.58283497246254)

- dre:100004503

- Up regulated genes

100004503(1.08159172673822)

- dre:100005893

- Up regulated genes

100005893(2.69284130859211)

- dre:393693

- Up regulated genes

393693\_1(8.42626553180885)

- dre:406860

- Up regulated genes

406860(3.03792201745619)

Close
